# Molecular dissection of effector mechanisms of *RAS*-mediated resistance to anti-EGFR antibody therapy

**DOI:** 10.18632/oncotarget.17438

**Published:** 2017-04-26

**Authors:** Stefan Kasper, Henning Reis, Sophie Ziegler, Silke Nothdurft, Andre Mueller, Moritz Goetz, Marcel Wiesweg, Jeannette Phasue, Saskia Ting, Sarah Wieczorek, Anna Even, Karl Worm, Michael Pogorzelski, Sandra Breitenbuecher, Johannes Meiler, Andreas Paul, Tanja Trarbach, Kurt Werner Schmid, Frank Breitenbuecher, Martin Schuler

**Affiliations:** ^1^ Department of Medical Oncology, West German Cancer Center, University Hospital Essen, University Duisburg-Essen, 45122 Essen, Germany; ^2^ Institute of Pathology, West German Cancer Center, University Hospital Essen, University Duisburg-Essen, 45122 Essen, Germany; ^3^ Department of General, Visceral und Transplantation Surgery, University Hospital Essen, University Duisburg-Essen, 45122 Essen, Germany; ^4^ German Cancer Consortium (DKTK), Partner Site University Hospital Essen, 45122 Essen, Germany; ^5^ Present address: Center for Tumor Biology and Integrative Medicine, Hospital Wilhelmshaven, 26389 Wilhelmshaven, Germany

**Keywords:** MAPK, PI3K/AKT, resistance, anti-EGFR antibody, colorectal cancer

## Abstract

Monoclonal antibodies targeting the epidermal growth factor receptor (EGFR), cetuximab and panitumumab, are a mainstay of metastatic colorectal cancer (mCRC) treatment. However, a significant number of patients suffer from primary or acquired resistance. *RAS* mutations are negative predictors of clinical efficacy of anti-EGFR antibodies in patients with mCRC. Oncogenic *RAS* activates the MAPK and PI3K/AKT pathways, which are considered the main effectors of resistance. However, the relative impact of these pathways in *RAS*-mutant CRC is less defined. A better mechanistic understanding of *RAS*-mediated resistance may guide development of rational intervention strategies. To this end we developed cancer models for functional dissection of resistance to anti-EGFR therapy *in vitro* and *in vivo*. To selectively activate MAPK- or AKT-signaling we expressed conditionally activatable RAF-1 and AKT in cancer cells. We found that either pathway independently protected sensitive cancer models against anti-EGFR antibody treatment *in vitro* and *in vivo*. RAF-1- and AKT-mediated resistance was associated with increased expression of anti-apoptotic BCL-2 proteins. Biomarkers of MAPK and PI3K/AKT pathway activation correlated with inferior outcome in a cohort of mCRC patients receiving cetuximab-based therapy. Dual pharmacologic inhibition of PI3K and MEK successfully sensitized primary resistant CRC models to anti-EGFR therapy. In conclusion, combined targeting of MAPK and PI3K/AKT signaling, but not single pathways, may be required to enhance the efficacy of anti-EGFR antibody therapy in patients with *RAS*-mutated CRC as well as in *RAS* wild type tumors with clinical resistance.

## INTRODUCTION

The epidermal growth factor receptor (EGFR) is constitutively activated either by somatic mutations or by overexpression in multiple cancer entities [1]. Overexpression of the receptor is associated with reduced overall survival and enhanced metastasis in patients with colorectal (CRC) or head and neck squamous cell cancer (HNSCC) [1–3]. Accordingly, the EGFR is attractive for therapeutic targeting by monoclonal antibodies or tyrosine kinase inhibitors (TKI). Large randomized clinical trials have confirmed the efficacy of the anti-EGFR antibodies cetuximab and panitumumab as monotherapy or in combination with chemo- and radiotherapy in patients with CRC and HNSCC [4–10]. The EGFR-TKIs gefitinib, erlotinib and afatinib have dramatically improved therapeutic options for patients with *EGFR*-mutated non-small cell lung cancer (NSCLC) [11–14]. However, primary and acquired resistances to therapies targeting the EGFR are a clinically relevant problem. Aberrant activation of the mitogen-activated protein kinase (MAPK) or the phosphatidylinositol 3- kinase/protein kinase B (PI3K/AKT) pathway which act downstream of the EGFR is implied in resistance to anti-EGFR therapies. Activation of both pathways may result from (i) oncogenic mutations of signaling mediators or (ii) cross activation by additional growth factor receptors. Several retrospective analyses confirmed that patients with CRC harboring mutations of *Kirsten rat sarcoma viral oncogene homolog* (*KRAS)*, *Neuroblastoma RAS viral oncogene homolog (NRAS)*, *v-Raf murine sarcoma viral oncogene homolog B (BRAF)* or *phosphatidylinositol-4,5-bisphosphate 3-kinase catalytic subunit alpha (PIK3CA)*, or loss of the phosphatase and tensin homolog (PTEN), a negative regulator of the PI3K/AKT pathway, associate with reduced response rates to anti-EGFR antibodies [9, 15–25]. *KRAS* or *PIK3CA* mutations are also negative predictors for efficacy of EGFR-TKI therapy in patients with NSCLC [26, 27]. Cross activation of EGFR downstream pathways by the hepatocyte growth factor receptor (c-MET) mediates resistance to EGFR-TKIs and anti-EGFR antibodies in NSCLC and CRC models [28, 29]. However, the relative impact of the PI3K/AKT and MAPK pathways on resistance to EGFR-targeting therapies is unclear. Extensive cross-talk between the MAPK and the PI3K/AKT pathway has been described [30–32]. Activation of *RAS* by growth factor receptor signaling nor by oncogenic mutation activates the rapidly accelerated fibrosarcoma family (RAF) but also PI3K. Extracellular signal–regulated kinases 1/2 (ERK1/2), which act downstream of RAF in the MAPK pathway, can activate the PI3K/AKT pathway at the level of tuberous sclerosis complex 1 and 2 (TSC1 and 2) or mammalian target of rapamycin complex 1 (mTORC1) [31]. In contrast, constitutively activated PI3K/AKT signaling negatively triggers the MAPK pathway by phosphorylation of inhibitory sites of RAF [32]. So far the exact molecular mechanisms how activation of these central pathways mediates resistance to anti-EGFR targeted therapy are unclear. Better understanding will help to develop therapeutic strategies that more patients can profit from EGFR-targeting drugs.

Against this background we established models to study the impact of isolated activation of the MAPK and PI3K/AKT pathways on the response to anti-EGFR therapy. In addition we correlated markers of pathway activation in tumor biopsies from patients with mCRC treated at the West German Cancer Center with their response to cetuximab.

We find that isolated activation of MAPK- or AKT-signaling equally mediates resistance to cetuximab *in vitro* and *in vivo*. While conditional activation of RAF-1 upregulated anti-apoptotic B-cell lymphoma-extra large (BCL-XL) and B-cell lymphoma 2 (BCL-2) proteins conditionally activated AKT stabilized anti-apoptotic myeloid leukemia cell differentiation protein 1 (MCL-1). In tumor samples from patients with mCRC biomarkers of MAPK activation strongly correlated with markers of PI3K/AKT activity, both in *KRAS* wild type and *KRAS*-mutated tumors. Pharmacologic inhibition of the MAPK and the PI3K/AKT cascade was most effective in sensitizing *RAS*-mutated CRC models to anti-EGFR antibody therapy. In conclusion, the MAPK and the PI3K/AKT pathways independently mediate resistance to anti-EGFR therapies.

## RESULTS

### Coactivation of the MAPK and PI3K/AKT pathways in *RAS*-mutated CRC

**Figure 1:**
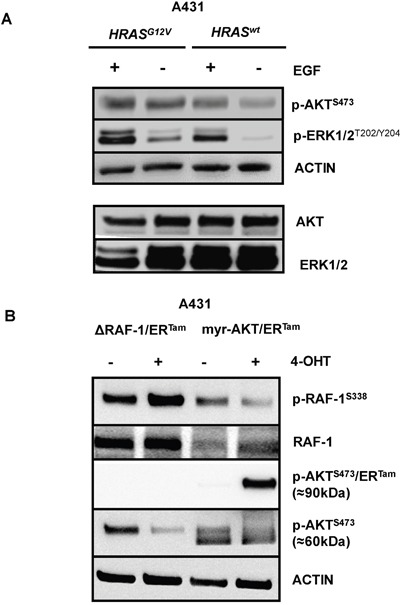
Coactivation of the MAPK and PI3K/AKT pathways in *RAS*-mutated EGFR-positive cancer cells **(A)** A431 cells were retrovirally transduced to stably express the oncogenic *RAS* mutant *HRAS^G12V^*. Constitutive and ligand-induced (EGF 10 ng/ml) phosphorylation of PI3K/AKT and MAPK signal transducers AKT and ERK1/2 in A431-HRAS^G12V^ cells or controls. ACTIN serve as control for equal loading. **(B)** A431-*RAS* wild type cells were retrovirally transduced to stably express a ΔRAF-1/ER^Tam^- or a myristoylated-AKT/ER^Tam^ (myr-AKT/ER^Tam^) construct. Phosphorylation of RAF-1 was strongly induced in A431-ΔRAF-1/ER^Tam^ cells and phosphorylation of myr-AKT/ER^Tam^ was strongly induced in A431-myr-AKT/ER^Tam^ cells by the addition of 4-hydroxytamoxifen (4-OHT).

Somatic *RAS* mutations are negative predictors of the efficacy of anti-EGFR antibodies in patients with mCRC. We have previously shown that oncogenic *RAS* mediates resistance by upregulation and stabilization of the anti-apoptotic protein BCL-XL [33]. As *RAS* signaling is coupled to the MAPK and the PI3K/AKT pathways we aimed to develop models for functional dissection of the relative contribution of these pathways to the RAS-mediated resistance phenotype of CRC. To this end we stably expressed *HRAS^G12V^* in the EGFR-positive, cetuximab-sensitive cancer cell lines A431 and Difi [33]. A431-*HRAS^G12V^*- and Difi-*HRAS^G12V^* cells exhibited higher levels of pERK1/2^T202/Y204^ and pAKT^S473^ than their *RAS^wt^* counterparts (Figure 1A and data not shown). This indicates co- or cross-activation of MAPK and PI3K/AKT signaling by oncogenic *RAS*, which is in line with previous observations [30–32].

### Activated MAPK and PI3K/AKT signaling confers resistance to anti-EGFR targeted therapy

To dissect the relative contribution of each pathway to resistance against anti-EGFR therapy, we stably expressed a ΔRAF-1/ER^Tam^- or a myristoylated-AKT/ER^Tam^ (myr-AKT/ER^Tam^) construct in *RAS* wild type A431 and Difi cancer cell lines. Both transgenes are conditionally activated by addition of hydroxytamoxifen (4-OHT) [34]. Functional transgene expression was confirmed by immunoblot analyses of phosphoepitopes indicating 4-OHT-induced ΔRAF-1/ER^Tam^- or myr-AKT/ER^Tam^ activation (Figure 1B and Supplementary Figure 1). Due the higher molecular weight of the myr-AKT/ER^Tam^ fusion construct (90kDa) the phosphorylated transgenic protein could be easily separated from endogenous AKT (60kDa). Interestingly, phosphorylation of endogenous RAF-1 was not increased in 4-OHT-treated A431-myr-AKT/ER^Tam^ cells, and phosphorylation of endogenous AKT was not enhanced in 4-OHT-treated A431-ΔRAF-1/ER^Tam^ cells. In fact, phosphorylation of these signaling mediators was rather reciprocally reduced, which might be explained by the activation of negative feedback regulation as suggested by Zimmermann and Moelling [35] (Figure 1B).

**Figure 2:**
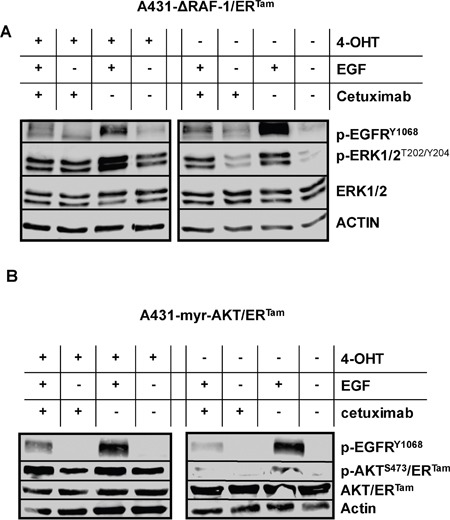
ΔRAF-1/ER^Tam^ and myr-AKT/ER^Tam^ restores EGFR downstream signaling in cetuximab treated cells A431-ΔRAF-1/ER^Tam^- **(A)** and A431-myr-AKT/ER^Tam^ **(B)** cells were incubated with 4-OHT, EGF (10 ng/ml) or cetuximab (1 μg/ml). **(A)** In the absence of 4-OHT, phosphorylation of EGFR and ERK was strongly induced by EGF. Cetuximab inhibited the ligand induced activation of EGFR downstream signaling. Upon pre-incubation with 4-OHT phosphorylation of ERK1/2 as marker of MAPK signaling was strongly induced, independently of incubation with EGF or cetuximab. **(B)** In the absence of 4-OHT, phosphorylation of EGFR and AKT/ER^Tam^ was strongly induced by EGF. Cetuximab inhibited the ligand induced activation of EGFR downstream signaling. Upon pre-incubation with 4-OHT phosphorylation of AKT/ER^Tam^ as marker of PI3K/AKT signaling was strongly induced, independently of incubation with EGF or cetuximab.

Next, we incubated both transgenic A431 cell lines with EGF, the monoclonal EGFR-antibody cetuximab, and the combination of both. In the absence of 4-OHT EGF dramatically induced the phosphorylation of EGFR, ERK1/2 and AKT indicating activation of the MAPK- and PI3K/AKT pathways (Figure 2A, 2B). In contrast, cetuximab reduced the activation of EGFR signaling. When A431-ΔRAF-1/ER^Tam^ cells were pre-incubated with 4-OHT markers of MAPK signaling were strongly activated, independently of incubation with EGF or cetuximab (Figure 2A). In line, 4-OHT pre-incubation of A431-myr-AKT/ER^Tam^ cells strongly induced markers of PI3K/AKT pathway activation (Figure 2B). Hence, our models were well suited for isolated functional analysis of either MAPK- or AKT-signaling (Figure 2A, 2B).

**Figure 3:**
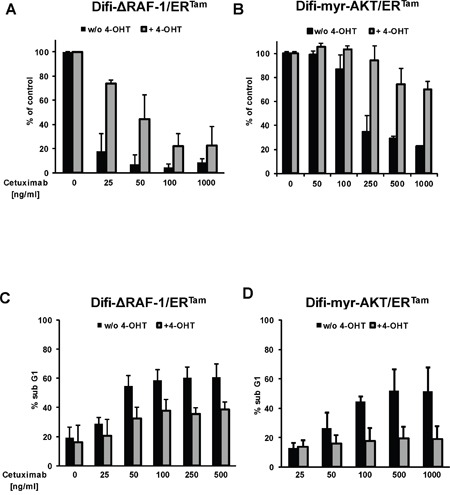
Activated MAPK and PI3K/AKT signaling confers resistance to anti-EGFR targeted therapy *in vitro* Clonogenic survival of Difi-ΔRAF-1/ER^Tam^ **(A)** and Difi-myr-AKT/ER^Tam^ **(B)** cells cultured in the presence of cetuximab with (gray columns) or without (black columns) 4-OHT for 10 days. Mean colony numbers (+ SD) normalized to medium control from three independent experiments are given. **(C, D)** Induction of apoptosis by cetuximab in Difi-ΔRAF-1/ER^Tam^ **(C)** and Difi-myr-AKT/ER^Tam^ cells **(D)** with (gray columns) or without (black columns) 4-OHT for 48h. Mean percentages (+ SD) of cells with subgenomic DNA content from three independent experiments are given.

**Figure 4:**
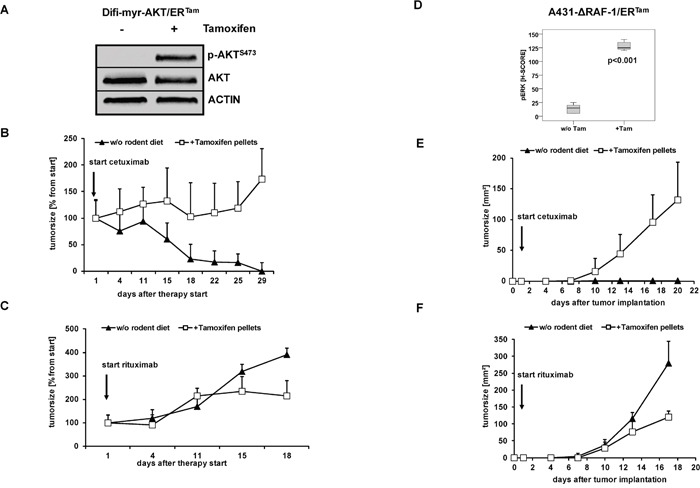
Activated PI3K/AKT and MAPK signaling protects tumors against anti-EGFR antibody-mediated cytotoxicity *in vivo* **(A)** Palpable flank tumors were established by subcutaneous injection of A431-myr-AKT/ER^Tam^ cells in NOD/SCID mice. Tumor-bearing mice were fed with diet with or without tamoxifen for transgene activation *in vivo* for one week. A strong phosphorylation of AKT as marker of PI3K/AKT signaling was detected in protein lysates from explanted tumors by immunoblot analyses. **(B, C)** Tumor growth following injection of Difi-myr-AKT/ER^Tam^ cells in NOD/SCID mice fed with (open boxes) or without (closed triangles) tamoxifen. After tumors were palpable (arrow), mice were treated biweekly with intraperitoneal injections of cetuximab (0.5 mg) **(B)** or rituximab (1 mg) **(C)**. Mean bidimensional tumor sizes (+ SD) of 5 mice per group are given. **(E, F)** NOD/SCID mice were fed with diet with or without tamoxifen for one week before A431-myr-ΔRAF-1/ER^Tam^ cells were subcutaneously implanted. The day after the tumor implantation mice were treated biweekly with intraperitoneal injections of cetuximab (1 mg) **(E)** or rituximab (1 mg) **(F)**. Tumor growth in NOD/SCID mice fed with (open boxes) or without (closed triangles) tamoxifen was measured bidimensional twice weekly. Mean bidimensional tumor sizes (+ SD) of 5 mice per group are given. **(D)** Palpable flank tumors of mice treated with rituximab were explanted and analyzed by immunhistochemistry. A strong phosphorylation of ERK1/2 was detected in explanted tumors of mice fed with tamoxifen diet.

Next, we analyzed the impact of conditional pathway activation on the response to anti-EGFR therapy. In the absence of 4-OHT both clinically approved anti-EGFR antibodies, cetuximab and panitumumab, effectively inhibited proliferation and clonogenic survival of Difi cancer cells *in vitro*. The addition of 4-OHT protected Difi-ΔRAF-1/ER^Tam^- and Difi-myr-AKT/ER^Tam^ cells against anti-EGFR antibody therapy *in vitro* (Figure 3A, 3B and Supplementary Figure 2). Moreover, 4-OHT-activated ΔRAF-1/ER^Tam^ and myr-AKT/ER^Tam^ protected Difi cells against cetuximab-induced apoptosis (Figure 3C, 3D). To study the impact of PI3K/AKT or MAPK pathway activation on the response to cetuximab in a host context, tumors were established by subcutaneous injection of A431 and Difi cells expressing the myr-AKT/ER^Tam^ or the ΔRAF-1/ER^Tam^ construct in non-obese diabetic severe combined immunodeficiency (NOD/SCID) mice. Mice were fed with diet with or without tamoxifen for transgene activation *in vivo*. Immunoblot analyses of explanted myr-AKT/ER^Tam^ tumors confirmed activation of myr-AKT/ER^Tam^ in tamoxifen-fed mice (Figure 4A). Explanted ΔRAF-1/ER^Tam^ tumors were immunohistochemically stained for pERK1/2. Tumors from tamoxifen-fed mice stained strongly positive for pERK1/2 (mean H-Score 129) compared to explanted tumors from mice without tamoxifen diet (mean H-Score 13, p<0.01; Mann-Whitney test) (Figure 4D and Supplementary Figure 3A). For further analyses of pathway activation by the myr-AKT/ER^TAM^ construct we selected mice bearing Difi tumors as they proved highly responsive to cetuximab *in vivo* [33]. NOD/SCID mice bearing palpable flank tumors were fed with or without tamoxifen diet. Two days after the initiation of tamoxifen diet, mice were treated by twice weekly intraperitoneal antibody injections of cetuximab (0.5 mg) or the anti-CD20 control antibody rituximab (1 mg), and tumor development was monitored. Cetuximab induced a dramatic shrinkage of established Difi-myr-AKT/ER^Tam^ tumors in mice not receiving tamoxifen diet. When myr-AKT/ER^Tam^ was activated by tamoxifen tumors were significantly protected against cetuximab (Figure 4B). No difference in tumor growth was observed between mice fed with or without tamoxifen diet which were treated with the control antibody rituximab (Figure 4C). To study the impact of pathway activation with the ΔRAF-1/ER^Tam^ construct *in vivo* we applied an “adjuvant treatment setting” and A431 cells as previously published [33]. Mice were fed with or without tamoxifen for one week. Intraperitoneal antibody injections with cetuximab or the anti-CD20 control antibody rituximab (1 mg twice weekly) were initiated one day after subcutaneous implantation of A431-ΔRAF-1/ER^Tam^ cells. Tumor development and survival were monitored. Cetuximab completely prevented the outgrowth of A431-ΔRAF-1/ER^Tam^ tumors in NOD/SCID mice fed without tamoxifen diet (Figure 4E). In contrast all tamoxifen-fed mice developed tumors despite cetuximab treatment, resulting in a dramatically inferior survival (p=0.002, log rank) (Figure 4E and Supplementary Figure 3B). Treatment with the control antibody rituximab failed to prevent development of A431-ΔRAF-1/ER^Tam^ tumors in mice fed with or without tamoxifen diet. Survival times were short in both groups irrespective of tamoxifen diet (p=0.317, log rank) (Figure 4F and Supplementary Figure 3B).

In conclusion, both signaling pathways, MAPK and PI3K/AKT, acting downstream of oncogenic *RAS* confer resistance of cancer cells to anti-EGFR antibody therapy *in vitro* and *in vivo*.

### Activated MAPK and PI3K/AKT signaling upregulates anti-apoptotic BCL-2 proteins

**Figure 5:**
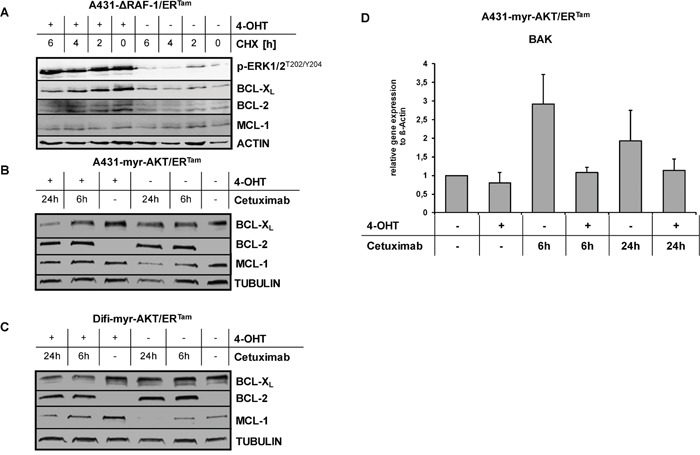
Upregulation of anti-apoptotic BCL2 proteins by activated MAPK and PI3K/AKT pathway **(A)** Expression of BCL-XL, BCL-2 and MCL-1 in A431-ΔRAF-1/ER^Tam^ cells with or without 4-OHT pre-incubation. To prevent new protein synthesis cells were treated with cycloheximide (CHX, 10 mg/mL) for indicated periods. **(B, C)** Expression of BCL-XL, BCL-2 and MCL-1 in A431-myr-AKT/ER^Tam^ **(B)** and Difi-myr-AKT/ER^Tam^ **(C)** cells treated with cetuximab (1 μg/ml) for indicated periods. **(D)** Relative *BAK* transcript level in A431-myr-AKT/ER^Tam^ cells treated with cetuximab (1 μg/ml) for indicated periods with or without 4-OHT. Expression levels of *BAK* were normalized to the housekeeping gene *beta-ACTIN*. Mean change (+ SD) of *BAK* transcript levels from three independent experiments are given.

Recently, we have shown that oncogenic *RAS* or activation of *RAS* downstream signaling mediates resistance by increased expression of anti-apoptotic BCL-2 proteins through transcriptional and posttranscriptional mechanisms [33, 36, 37]. BCL-2-type proteins protect against apoptosis by preventing permeabilization of the mitochondrial outer membrane [38]. Against this background, we analyzed the expression of the anti-apoptotic BCL-2 family members BCL-2, BCL-XL and MCL-1 in our cancer models with conditional pathway activation. Treating A431-ΔRAF-1/ER^Tam^ cells with 4-OHT induced the expression of BCL-XL and BCL-2, but not MCL-1. Blocking protein synthesis with cycloheximide revealed significantly enhanced stability of BCL-XL and BCL-2, suggesting a posttranslational mechanism (Figure 5A). In contrast, 4-OHT treatment of A431-myr-AKT/ER^Tam^- and Difi-myr-AKT/ER^Tam^ cells had no impact on the expression of BCL-XL or BCL-2, but strongly induced and stabilized MCL-1 (Figure 5B, 5C) [36]. Cetuximab decreased the expression of MCL-1 in a time dependent manner, which was delayed by activation of myr-AKT/ER^Tam^. In contrast, no change in BCL-XL levels was seen (Figure 5B, 5C). Interestingly, basal BCL-2 expression was very low but was strongly induced by the addition of cetuximab in both cell lines. As BCL-2 levels were increased following 4-OHT in the A431-ΔRAF-1/ER^Tam^ model (Figure 5A), this observation might be explained by AKT-induced compensatory feed-back activation of MAPK signaling (Figure 5B, 5C). In addition to anti-apoptotic BCL-2 family proteins, programmed cell death is regulated by pro-apoptotic family members. In this process the BH1-2-3 proteins bcl-2 homologous antagonist/killer (BAK) and bcl-2-like protein 4 (BAX) are essential mediators of mitochondrial outer membrane permeabilization [38, 39]. In line, cetuximab treatment induced the RNA expression of pro-apoptotic *BAK* in A431-myr-AKT/ER^Tam^ cells in the absence of 4-OHT. This effect was completely abolished by addition of 4-OHT (Figure 5D).

Taken together, conditional activation of RAF-1 signaling increased the expression of anti-apoptotic BCL-XL and BCL-2, whereas activation of AKT signaling stabilized anti-apoptotic MCL-1 and prevented the expression of pro-apoptotic *BAK*. The endpoint of both events is protection of cancer cells to apoptosis executed via the intrinsic pathway of caspase activation [39].

### Biomarkers of MAPK and PI3K/AKT pathway activation correlate with outcome in cetuximab-treated colorectal cancer patients

To corroborate our preclinical findings and to specifically study the impact of activated MAPK and PI3K/AKT signaling on cetuximab-based therapy we analyzed a retrospective cohort of mCRC patients. Formalin-fixed, paraffin-embedded (FFPE) surplus tumor tissue obtained at diagnostic biopsy or resection was available for analysis from 39 patients with histologically confirmed CRC, who had been treated with cetuximab between 2004 and 2007. This cohort is particularly suited to compare the impact of oncogene mutations and biomarkers of pathway activation, as these patients were treated before cetuximab therapy was restricted to patients with *RAS* wild typic tumors [9]. Patients’ characteristics are summarized in Supplementary Table 1. Tissue sections were immunohistochemically stained for pERK1/2, pAKT and pp70S6K1 as markers of pathway activation, and tumor-derived DNA was analyzed for mutational status of *KRAS* exon 2 and *BRAF* exon 15 by amplicon sequencing.

*KRAS* exon 2 mutations were detected in 14 patients (39%), and *BRAF* exon 15 mutations were detected in 3 patients (9%). Almost 50% of tumors stained negatively or only slightly positive for pAKT^S473^ and thus were grouped as low expressers (0+ and 1+). The other 50% of tumors stained moderately or highly positive for pAKT and were grouped as high expressers (2+ and 3+) (Supplementary Table 2 and Supplementary Figure 4). Inhomogeneous staining patterns were detected for pERK1/2 and p-Ribosomal protein S6 kinase beta-1 (pp70S6K1). Thus, the absolute numbers of IHC-positive cells were counted instead of categorizing into low and high expressers. Samples were subsequently dichotomized according to IHC-positive cell counts below and above the median of the entire population (≤median; >median) (Supplementary Table 2 and Supplementary Figure 4). As expected detection of pAKT correlated with pp70S6K1 positivity as both proteins are activated by canonical PI3K/AKT signaling (Table 1A). In addition, a positive correlation was observed between pERK1/2 and pAKT positivity, and between pERK1/2 and pp70S6K1. This is in line with cross- or co-activation of MAPK and PI3K/AKT signaling in human CRC (Table 1A). Interestingly, this co-activation was equally observed in samples with wild-typic and mutated *KRAS* (exon 2) (Table 1B). As additional mutations in *KRAS* exons 3 and 4 and *NRAS* exon 2 to 4 are observed in mCRC and were described as negative predictors for the efficacy of anti-EGFR antibodies, we reanalyzed residual samples for these additional mutations. In 23 out of 39 samples the “*all RAS* and *BRAF”* mutational analyses was feasible, and in total 17 *RAS* or *BRAF* mutations were detected. The baseline characteristics of these 23 comprehensively analyzed patients were similar to the entire population (data not shown). Similar overall correlations between pAKT, pp70S6K1 and pERK positivity were observed in this subpopulation (Supplementary Table 3A). However, this was entirely carried by the subgroup of patients with mCRC mutated in *RAS* or *BRAF* (Supplementary Table 3B).

**Table 1A:** Study population (N=39)

All pts	pAKT^S473^ low	pAKT^S473^ high	pERK1/2^T202/Y204^ ≤ median	pERK1/2^T202/Y204^ > median
**pp70S6K1^T389^ < median**	13	8	18	3
**pp70S6K1^T389^ > median**	6	12	3	15
	p = 0.075, chi-square;odds ratio (95% CI): 3.250 (0.870-12.137)		p < 0.0001, chi-square;odds ratio (95% CI): 30.000 (5.261-171.062)	
**pERK1/2^T202/Y204^ ≤ median**	14	7		
**pERK1/2^T202/Y204^ > median**	5	13		
	p = 0.015, chi-square;odds ratio (95% CI): 5.200 (1.317-20.539)			

**Table 1B:** *KRAS* exon 2 population (N=36)

	*KRAS wt*		*KRAS mut*		*KRAS wt*		*KRAS mut*	
	pp70S6K1^T389^ ≤ median	pp70S6K1^T389^ > median	pp70S6K1^T389^ ≤ median	pp70S6K1^T389^ > median	pAKT^S473^ low	pAKT^S473^ high	pAKT^S473^ low	pAKT^S473^ high
**pERK1/2^T202/Y204^≤ median**	7	3	9	0	8	2	5	4
**pERK1/2^T202/Y204^> median**	2	10	1	4	4	8	0	5
	p = 0.011, chi-square;odds ratio (95% CI): 11.667 (1.527-89.121)		p = 0.001, chi-square;odds ratio (95% CI): 5.000 (0.866-28.861)^1^		p = 0.029, chi-square; odds ratio (95% CI): 8.000 (1.127-56.793)		p = 0.038, chi-square;odds ratio (95% CI): 0.444 (0.214-0.923)^2^	

^1^For pp70S6K1^T389^ ≤ median cohort; ^2^for pAKT^S473^ high cohort.

To validate these findings a second, independent cohort of 88 mCRC patients was immunohistochemically analyzed for expression of pAKT, pERK1/2, pp70S6K1 and the phosphatase PTEN, a negative regulator of the PI3K/AKT pathway. An independent scoring system (H-Score) was applied, and biomarker positivity was correlated with the mutational status of *KRAS*, *NRAS*, *BRAF* and *PIK3CA* (Supplementary Tables 4A, 4B). Positive correlation between markers of MAPK and PI3K/AKT pathway activation was observed, which confirmed the findings obtained in the first patient cohort (Table 2A). In this validation cohort, *RAS* mutations were detected in 33% of patients, which associated with positivity for pERK1/2 as biomarker for MAPK pathway activation (Table 2B). *PI3KCA* mutations were found in 8% of patients which strongly correlated with positivity for pAKT and pp70S6K as biomarkers for PI3K/AKT pathway activation (Table 2B).

**Table 2A:** Marker correlation

All pts	pAKT^S473^	pERK1/2^T202/Y204^	pp70S6K1^T389^	PTEN^loss^
**pp70S6K1^T389^**				
p-value*	0.015^#^	0.020^#^	n.a.	0.018^#^
**pERK1/2^T202/Y204^**				
p-value*	0.207	n.a.	0.020^#^	0.012^#^
**pAKT^S473^**				
p-value*	n.a.	0.207	0.015^#^	0.121^#^

n.a.: not applicable; *Spearman-rho; ^#^significance p < 0.05

**Table 2B:** *RAS*, *BRAF*, *PIK3CA* and marker correlation

	pAKT^S473^	pERK1/2^T202/Y204^	pp70S6K1^T389^	PTEN^loss^
**All *RAS* mutation^1^**				
p-value*	0.340	0.035^#^	0.175	0.168
***BRAF* mutation^2^**				
p-value*	0.406	0.161	0.219	0.275
**all *RAS* or *BRAF* mutation^1,2^**				
p-value*	0.392	0.111	0.097^§^	0.109
***PIK3CA* mutation^3^**				
p-value*	0.001*	0.055^§^	0.496	0.214

n.a.: not applicable; ^1^KRAS exon 2-4, NRAS exon 2-4; ^2^BRAF exon 15; ^3^*PIK3CA* exon 10 and 21; *Spearman-Rho; ^#^denotes significance (p < 0.05); §trend (p < 0.1).

### Biomarkers of MAPK and PI3K/AKT pathway activation negatively predict response to cetuximab in patients with mCRC

**Figure 6:**
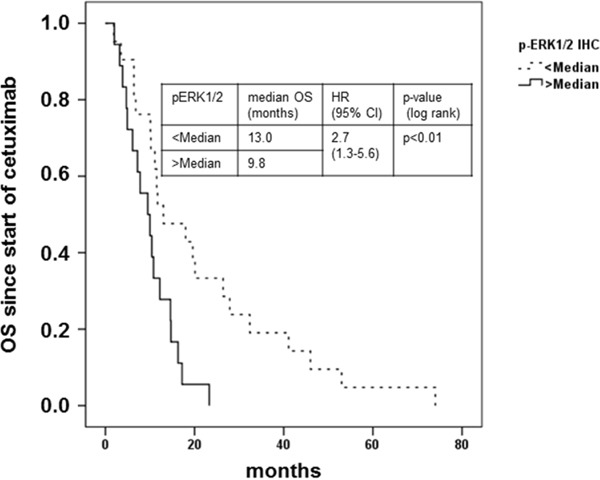
Clinical outcome of patients with metastatic colorectal cancer treated with cetuximab in combination with irinotecan in relation to pERK1/2^T202/Y204^ expression Kaplan-Meier plot of overall survival (OS) from start of treatment with cetuximab in combination with irinotecan of patients with mCRC with immunohistochemical pERK1/2^T202/Y204^ stainig intensity below (dashed line) or above median (solid line). Patients with tumors stained for pERK1/2^T202/Y204^ below the median demonstrated a prolonged OS (13.0 vs. 9.8 months, HR 2.7; 95% CI 1.3-5.6); p < 0.01 log rank).

Next, we correlated *KRAS* and *BRAF* mutation status and markers of downstream pathway activation with the clinical outcome of cetuximab-based therapy (Table 3 and Supplementary Table 3C-3F for “*all RAS/BRAF”* cohort). Patients with *KRAS* exon 2 wild-typic and “all *RAS”* wild-typic tumors, respectively, had a numerically higher ORR (Table 4A and Supplementary Table 3D). Formal statistical significance was not established due to the limited sample size. Interestingly, neither median Progression free survival (PFS) nor median Overall survival (OS) differed between patients with *KRAS* exon 2 wild-typic or “*all RAS”* wild-typic and mutated tumors (Table 4B, 4C and Supplementary Table 3E, 3F). No patient with a *BRAF*-mutated tumor objectively responded to cetuximab-based therapy. Median PFS was significantly reduced whereas median OS did not differ (Table 4A–4C and Supplementary Table 3E, 3F). Patients with tumors staining low for pERK1/2^T202/Y204^ had better ORR, longer median PFS and longer median OS as compared to patients with high pERK1/2^T202/Y204^ staining (Table 4A–4C, Supplementary Table 3D-3F and Figure 6). This finding was reproducible when patients were grouped by *KRAS exon 2/BRAF or “all RAS/BRAF”* mutational status. However, formal statistical significance was not established in the “*all RAS”* population due to limited sample size. Also, ORR of patients with low staining for pAKT or pp70S6K1 was higher than in patients with high staining for these biomarkers of AKT pathway activation, but PFS did not differ (Table 4A, 4B and Supplementary Table 3D-3F). The OS was numerically longer in patients with low pAKT or pp70S6K1 staining (Table 4C and Supplementary Table 3F and Supplementary Figure 5). In summary, these findings in mCRC patients support the negative predictive value of biomarkers of MAPK and PI3K/AKT pathway activation for response to cetuximab-based therapy.

**Table 3A:** Efficacy data (study population; N=39)

	N	%
ORR	7	18
CR	0	0
PR	7	18
SD	22	56
PD	10	26
Median PFS	3.5 months (1.9-10.1)	
Median OS since start of cetuximab	11.1 months (1.9-74.0)	

**Table 4A:** ORR

Marker	ORR (%)	Odds ratio (95% CI)	p-value
All patients	18		
*KRAS wt* vs mut*	22 vs 14	1.7 (0.2-10.6)	0.43
*BRAF wt* vs *mut*^#^	19 vs 0	0.7 (0.1-8.2)	0.54
pERK1/2^T202/Y204^ < median vs > median	29 vs 6	6.8 (0.7-63.1)	0.07
pAKT^S473^ 0+/1+ vs 2+/3+	31 vs 5	8.7 (0.9-81.9)	0.04
pp70S6K1^T389^ < median vs > median	23 vs 11	2.5 (0.4-14.8)	0.27
*KRAS wt/BRAF wt* andpERK1/2^T202/Y204^ < median vs > median	40 vs 14	4.0 (0.3-40.1)	0.25
KRAS mut* or *BRAF mut^#^* andpERK1/2^T202/Y204^ < median vs > median	22 vs 0	2.1 (1.2-3.7)	0.16
*KRAS wt/BRAF wt* andpAKT^S473^ 0+/1+ vs 2+/3+	40 vs 14	4.0 (0.3-47.1)	0.25
KRAS mut* or *BRAF mut^#^* andpAKT^S473^ 0+/1+ vs 2+/3+	33 vs 0	3.8 (1.6-8.7)	0.04
*KRAS wt/BRAF wt* andpp70S6K1^T389^ < median vs > median	43 vs 20	3.0 (0.3-25.9)	0.31
KRAS mut* or *BRAF mut^#^* andpp70S6K1^T389^ < median vs > median	17 vs 0	1.5 (1.0-2.1)	0.33

*exon 2; ^#^exon 15.

**Table 4B:** PFS

Marker	Median PFS (months)	HR (95% CI)	p-value(log rank)
All patients	3.5		
*KRAS wt* vs mut*	3.5 vs 4.0	0.8 (0.4-1.6)	0.58
*BRAF wt* vs *mut*^#^	3.5 vs 1.4	4.2 (1.1-15.7)	0.03
pERK1/2^T202/Y204^ < median vs > median	4.0 vs 3.1	1.8 (0.9-3.6)	0.09
pAKT^S473^ 0+/1+ vs 2+/3+	3.5 vs 4.1	0.7 (0.4-1.4)	0.32
pp70S6K1^T389^ < median vs > median	3.5 vs 3.7	0.9 (0.5-1.8)	0.91
*KRAS wt/BRAF wt* andpERK1/2^T202/Y204^ < median vs > median	3.6 vs 3.0	1.9 (0.7-5.5)	0.22
KRAS mut* or *BRAF mut^#^* andpERK1/2^T202/Y204^ < median vs > median	4.0 vs 2.5	1.7 (0.6-4.7)	0.31
*KRAS wt/BRAF wt* andpAKT^S473^ 0+/1+ vs 2+/3+	3.0 vs 5.0	0.9 (0.3-2.4)	0.76
KRAS mut* or *BRAF mut^#^* andpAKT^S473^ 0+/1+ vs 2+/3+	4.0 vs 3.3	0.8 (0.3-2.4)	0.67
*KRAS wt/BRAF wt* andpp70S6K1^T389^ < median vs > median	3.5 vs 4.1	1.0 (0.4-2.7)	0.97
KRAS mut* or *BRAF mut^#^* andpp70S6K1^T389^ <median vs > median	3.5 vs 2.8	0.9 (0.3-2.8)	0.89

*exon 2; ^#^exon 15.

**Table 4C:** OS

Marker	Median OS (months)	HR (95% CI)	p-value(log rank)
All patients	11.1		
*KRAS wt* vs mut*	11.3 vs 10.6	0.8 (0.4-1.6)	0.55
*BRAF wt* vs *mut*^#^	10.2 vs 10.8	1.5 (0.5-5.2)	0.51
pERK1/2^T202/Y204^ < median vs > median	13.0 vs 9.8	2.7 (1.3-5.6)	<0.01
pAKT^S473^ 0+/1+ vs 2+/3+	12.2 vs 10.4	1.5 (0.8-2.9)	0.22
pp70S6K1^T389^ < median vs > median	11.1 vs 10.4	1.2 (0.6-2.3)	0.52
*KRAS wt/BRAF wt* andpERK1/2^T202/Y204^ < median vs > median	19.6 vs 6.1	4.2 (1.2-15.1)	0.02
KRAS mut* or *BRAF mut^#^* andpERK1/2^T202/Y204^ < median vs > median	11.5 vs 7.8	3.2 (1.0-9.9)	0.03
*KRAS wt/BRAF wt* andpAKT^S473^ 0+/1+ vs 2+/3+	14.7 vs 9.5	2.0 (0.7-6.0)	0.20
KRAS mut* or *BRAF mut^#^* andpAKT^S473^ 0+/1+ vs 2+/3+	10.2 vs 10.8	1.0 (0.4-2.8)	0.97
*KRAS wt/BRAF wt* andpp70S6K1^T389^ < median vs > median	19.6 vs 9.5	1.4 (0.5-4.0)	0.50
KRAS mut* or *BRAF mut^#^* andpp70S6K1^T389^ < median vs > median	10.8 vs 7.8	1.9 (0.6-5.9)	0.25

*exon 2; ^#^exon 15.

### Combined pharmacologic targeting of MAPK and PI3K/AKT pathways resensitizes EGFR-positive cancer cells to anti-EGFR therapies

**Figure 7:**
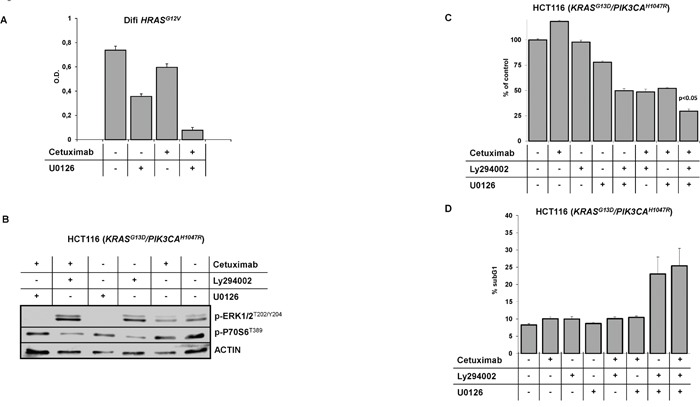
Combined pharmacologic targeting of MAPK and PI3K/AKT signaling resensitizes EGFR positive cells to anti-EGFR therapies **(A)** Proliferation of Difi-HRAS^G12V^ cells grown in the presence of cetuximab (100 ng/ml), the MEK inhibitor U0126 (1 μM) or in combination. Mean values (± SD) of three independent MTT assays. **(B)** Immunoblot analysis of HCT116 which harbors an endogenous *KRAS exon 2* and an additional *PIK3CA exon 20* mutation treated with cetuximab (20 μg/ml), the PI3K inhibitor Ly294002 (10 μM), the MEK inhibitor U0126 (1 μM) and in combination. Inhibition of constitutive phosphorylation of MAPK and PI3K/AKT signal transducers ERK1/2 and p70S6 by the pharmacological inhibitors Ly294002 and U0126, respectively. **(C)** Proliferation of HCT116 cells grown in the presence of cetuximab (20 μg/ml), the PI3K inhibitor Ly294002 (10 μM), the MEK inhibitor U0126 (1 μM) and in combination. Mean values (± SD) of three independent MTT assays. **(D)** Fraction of apoptotic HCT116 cells with subgenomic DNA content (sub-G1) following treatment with cetuximab (20 μg/ml], Ly294002 (15 μM), U0126 (1 μM) and in combination (mean ± SD of three independent experiments).

It is expected that rational combination of signaling inhibitors may delay or circumvent resistance, which is frequently encountered with targeted mono-therapies. This is exemplified by the recent approval of combined *BRAF* and MEK inhibition for therapy of patients with metastatic *BRAF*-mutated malignant melanoma [40–43]. In *BRAF*-mutated mCRC the rational use of *BRAF* inhibitors in combination with anti-EGFR targeting drugs has shown promising efficacy in preclinical models and in early clinical trials [44–46]. To explore whether combined MAPK and/or PI3K/AKT pathway inhibition would overcome *RAS*-mediated resistance to cetuximab we studied Difi-*HRAS^G12V^* cells and HCT116 cells, which harbor an endogenous *KRAS exon 2* mutation and an additional *PIK3CA exon 20* mutation. First, we tested the combination of cetuximab with the MEK inhibitor U0126 in Difi-*HRAS^G12V^* cells. U0126 inhibited the constitutive as well as EGF-induced phosphorylation of ERK1/2, whereas cetuximab did not inhibit ERK1/2 phosphorylation (Supplementary Figure 6). In line, cetuximab only marginally inhibited the proliferation of Difi-*HRAS^G12V^* cells. In contrast, U0126 strongly suppressed the proliferation of Difi-*HRAS^G12V^* cells. The combination of U0126 and cetuximab acted synergistically and completely inhibited proliferation of Difi-*HRAS^G12V^* cells (Figure 7A).

Next, we combined inhibitors of PI3K (Ly294002) and MEK (U0126) in the HCT116 model. HCT116 cells exhibit phosphorylation of ERK1/2 and p70S6K1 ribosomal protein, indicating constitutive activation of MAPK and PI3K/AKT signaling (Figure 7B). Treatment with cetuximab only slightly inhibited the phosphorylation of ERK1/2 and S6 ribosomal protein. Ly294002 markedly inhibited the phosphorylation of S6 ribosomal protein whereas phosphorylation of ERK1/2 was induced, arguing for compensatory feed-back activation. Treatment with U0126 completely prevented ERK1/2 phosphorylation. Treatment of HCT116 cells with cetuximab or the PI3K inhibitor Ly294002 alone failed to inhibit proliferation, while the MEK inhibitor U0126 had some anti-proliferative activity (Figure 7C). Interestingly the combination of Ly294002 with U0126 or the combination of one of the inhibitors with cetuximab dramatically reduced cell proliferation. The combination of all three compounds had the strongest effect with a nearly complete inhibition of proliferation. Neither treatment with cetuximab, Ly294002 or U0126 alone nor the combination of cetuximab with one of the inhibitors induced apoptosis in HCT116 cells (Figure 7D). In contrast, the combination of Ly294002 and U0126 markedly induced apoptosis. This effect could be further enhanced by the addition of cetuximab.

In conclusion, our preclinical and clinical data provide a strong argument to combine inhibitors of the MAPK and the PI3K/AKT pathways to overcome resistance to cetuximab in *RAS*-mutated colorectal cancer.

## DISCUSSION

The monoclonal anti-EGFR antibodies cetuximab and panitumumab are effective as monotherapy or in combination with chemotherapy in patients with mCRC [4–6, 9, 10]. In addition cetuximab is also approved in combination with radiotherapy for patients with locally advanced HNSCC ineligible for platinum based chemotherapy or in combination with platinum in the recurrent or metastatic setting [7, 8]. However, not all patients respond to this targeted therapy or acquire resistance. In patients with mCRC activating mutations of the *RAS, BRAF and PIK3CA* oncogenes have been identified as key predictors of primary resistance in comprehensive biomarker-analyses of large clinical trials [9, 15–25]. Additional aberrations associated with resistance to anti-EGFR antibody therapy in mCRC comprise amongst others amplification, mutations or overexpression of the human epidermal growth factor receptor 2 (HER2) and HER3, the hepatocyte growth factor receptor (c-MET), the platelet-derived growth factor receptor A (PDGFRA) and the fibroblast growth factor receptor 1 (FGFR1) [55, 56]. While these biomarkers which can be readily assessed in tumor biopsies indicate a clinical resistance phenotype, the actual mechanisms how resistance is mediated is less well defined. Current thinking implies deregulated intracellular signal transduction pathways as the main effectors of primary and acquired resistance to anti-EGFR antibody therapy. In particular the MAPK and PI3K/AKT pathways, which are activated by most growth factor receptor tyrosine kinases, are considered. However, these pathways do not act in linear, monodirectional ways but are closely interconnected with each other and additional signaling pathways. Hence, a clinical resistance phenotype has to be appreciated as the output of a complex interplay of external and internal signaling cues that are integrated by the cancer cell. Further understanding of such cross-talk and the exact molecular mechanisms how pathway activation mediates resistance is necessary to develop more precise therapeutic strategies breaking resistance to anti-EGFR antibodies.

Using transgenic expression of conditionally activatable activators of canonical MAPK and PI3K/AKT signaling we here demonstrate *in vitro* and in relevant *in vivo* models that either pathway is independently capable of mediating resistance to EGFR blockade. However, enforced expression of *RAS* mutants simultaneously activates both pathways, which is in line with cross-activation as previously described [30–32]. The clinical relevance of this observation is supported by our biomarker study of two independent cohorts of patients with mCRC, which revealed a strong correlation between markers of signal transduction pathway activation in primary tumor samples. Interestingly, an association of positivity for biomarkers of activated MAPK or PI3K/AKT signaling with lower likelihood of response to cetuximab and reduced overall survival from start of cetuximab treatment was found in patients with mCRC that was independent of the *RAS* or *BRAF* mutational status. This observation underscores the importance of pathway activation as mechanistic basis of resistance, which may result from somatic mutations or *RAS* or *BRAF* proto-oncogenes but also other causes. In the light of the limited sample size of our two clinical cohorts, these findings, however, require validation in larger, independent cohorts of patients with mCRC treated with anti-EGFR antibodies.

Conceptually our findings may have implications on therapeutic strategies to overcome resistance to cetuximab or panitumumab. In our preclinical models combined pharmacological modulation of the MAPK and the PI3K/AKT pathway by specific small molecule inhibitors sensitized *RAS*-mutated cancers to cetuximab. Clinical translation of these findings may be challenging with the toxicities of currently available inhibitors of MEK and PI3K. However, novel compounds specifically targeting ERK and specific subtypes of the RAF and PI3K kinases effective combination therapies may become clinically feasible [59, 60]

Deregulated growth factor signaling frequently impacts on the regulation of apoptotic cells death. In particular, high expression of anti-apoptotic proteins of the BLC-2 family is observed over several cancer entities, which is in line with our present findings in cancer models. Activation of the MAPK pathway resulted in stabilization of BCL-XL and BCL-2, whereas activation of the PI3K/AKT pathway resulted in an induction of MCL-1. We have previously identified BCL-XL upregulation as a main effector in preventing *RAS* mutant CRC cell lines from anti-EGFR antibody induced apoptosis, and in CD20-positive B-cell Non-Hodgkin Lymphoma (B-NHL) cells from rituximab-induced apoptosis [33, 57]. MCL-1 was previously identified by our group and others as mediator of resistance against cytotoxic drugs in acute myeloid leukemia (AML) but also against antigen-specific cytotoxic T-cells in solid tumor models [36, 58]. Mechanistically, these BCL-2 type proteins inhibit apoptosis by binding of the pro-apoptotic proteins BAX and BAK to prevent permeabilization of the mitochondrial outer membrane and subsequent caspase activation [38, 39]. In our most sensitive cancer models cetuximab induced apoptosis *in vitro* and shrinkage of established tumors *in vivo*. Interestingly, this associated with cetuximab-induced downregulation of MCL-1 and transcriptional induction of pro-apoptotic BAK, and both events were prevented by conditional activation of AKT signaling. We previously demonstrated that modulation of BCL-2 type proteins by pharmacological inhibitors or by siRNA sensitized tumor cells to different therapeutic strategies including cytotoxic drugs, immunotherapies and monoclonal antibodies [33, 36, 57, 58]. Thus, these molecular effectors of treatment resistance mediated by aberrant MAPK and PI3K/AKT pathway activation are additional targets of interest strategies to improve EGFR-directed therapies.

## MATERIALS AND METHODS

### Cell lines and reagents

The human EGFR-positive cancer cell lines A431 and the colorectal cancer cells HCT116 harboring an endogenous *KRAS exon 2* and a *PIK3CA exon 20* mutation were obtained from DSMZ (Braunschweig, Germany). Difi cells were obtained from R. Coffey (Nashville, Tennessee). All cells were cultured in DMEM supplemented with 10% fetal bovine serum (FBS, PAA, Coelbe, Germany), L-glutamine, penicillin and streptomycin (Invitrogen, Frankfurt, Germany). Stable expression of *KRAS*^G12V^ or *HRAS^G12V^* was achieved by retroviral transduction as described previously [47]. The myristoylated-AKT/ER^Tam^ (myr-AKT/ER^Tam^) construct kindly provided by J. Downward cloned into the retroviral vector plasmid pQCxIP (Clonetech) and the ΔRAF-1/ER^Tam^ construct subcloned into the retroviral pBabePuro vector kindly provided by S. Cook were stably expressed as described previously [36]. Clinical grade cetuximab (Erbitux, Merck Serono, Darmstadt, Germany) and rituximab (Mabthera, Roche, Grenzach-Wyhlen, Germany) were purchased from the pharmacy of the University Hospital Essen; U0126, Ly294002, 4-hydroxytamoxifen (4-OHT) were purchased from Sigma (Deisenhofen, Germany).

The following primary antibodies were used for immunoblotting following standard protocols: AKT1/2 (H-136), BCL-2 (C2) (all from Santa Cruz Biotechnology, Santa Cruz, CA), phospho-ERK1/2^T204Y204^, ERK1/2, phospho-AKT^S473^, BCL-XL (54H6), EGFR, phospho-EGFR^Y1068^, phospho-S6 ribosomal protein (all from Cell Signaling Technology, Danvers, MA), actin (C4, ICN, Irvine, CA), MCL-1 (Epitomics, Burlingame, CA).

### Gene expression analysis

For RNA expression analysis, total RNA was isolated (High Pure RNA Isolation Kit, Roche Diagnostics, Mannheim, Germany) and reversely transcribed into cDNA (Transcription High Fidelity cDNA Synthesis Kit, Roche Diagnostics, Mannheim, Germany) following the manufacturer's instructions. Quantitative RT-PCR analysis was performed on a LC480 instrument using SYBR Green 1 Master chemistry (Roche Diagnostics, Mannheim, Germany) and primers for human *BAK*: 5′-AACCGACGCTATGACT-3′, 5′-TCGTACCACAAACTGGC-3′ and human *A*CTIN: 5′-TCA GCT GTG GGG TCC TGT-3′, 5′-GAA GGG ACA GGC AGT GAG-3′ as previously described [33].

### Animal models

All animal studies were conducted in compliance with institutional guidelines and German Animal Protection Law, and were approved by the responsible regulatory authority (Landesamt für Natur, Umwelt und Verbraucherschutz Nordrhein-Westfalen, Az. G969/08). NOD/SCID mice (Charles River Laboratories, France) received single subcutaneous flank injections of 2×10^6^ A431 or 1×10^7^ Difi cells suspended in 200 μl saline. Tamoxifen rodent diet (400 mg/kg) was obtained by Harlan Teklad (Teklad CRD Tam 400/CreER, irradiation by 21 kGy/min). Animals were monitored for tumor development twice weekly, and tumor growth was bidimensionally quantified using a caliper. Antibodies were dissolved in 200 μl saline and administered as biweekly intraperitoneal injections. Protein lysates of representative tumors were prepared as described previously and analyzed by Immunoblotting [36]. After mice were scarified, tumors were explanted and fixed over night in 4% formalin. Sections were subjected to hematoxilin & eosin staining and immunohistochemical analyses following diagnostic protocols of the Institute of Pathology. The pERK1/2^T202/Y204^ staining was categorized according to the H-Score as described previously [53]. For statistical analysis, the Mann-Whitney test was used.

### Cellular assays

For clonogenic survival analysis, 5×10^3^ A431 or Difi cells were seeded in 6-well plates in the presence of the indicated antibodies or pharmacological inhibitors. Following incubation for 7 to 14 days colonies were fixed with ethanol (70% v/v) for 30 minutes, stained with Coomassie brilliant blue and automatically counted using an Infinity-100 System and the Vision Capt software (Vilber Lourmat, Eberhardzell, Germany). Proliferation was quantified by means of the MTT assay according to the manufacturer's instruction (Roche, Mannheim, Germany). Apoptosis was quantified by flow cytometric determination of cells with subgenomic DNA content following hypotonic lysis and staining with propidium iodide as previously described [44]. All results were obtained from at least three independent experiments.

### Analysis of primary tumor samples

Clinical data and surplus tumor specimens were retrieved from 39 patients with metastatic CRC. All patients were heavily pretreated and had a documented disease progression before initiation of irinotecan based chemotherapy. Subsequently, patients were switched to irinotecan in combination with cetuximab as established by the phase III “Bond”-trial [4]. All patients except two received irinotecan and cetuximab on a weekly schedule, whereas two patients received irinotecan every three weeks and cetuximab weekly. Baseline characteristics are summarized in Supplementary Table 1. Overall response rate (ORR) was determined by RECIST 1.1 [48]. Differences between the groups were calculated using the chi-square test and odds ratios (OD). Progression free survival (PFS) was defined as time from start of irinotecan and cetuximab to progressive disease or death, overall survival (OS) was defined as time from start of therapy to death. For statistical analyses, univariate Cox proportional hazards and log rank tests were used. Tissue microarrays were prepared from formalin-fixed, paraffin-embedded (FFPE) tumor samples. Sections were subjected to hematoxilin & eosin staining and immunohistochemical analyses following diagnostic protocols of the Institute of Pathology. The pAKT^S473^ staining was categorized into negative (0+), weak (1+), moderate (2+) and strong (3+) [49]. For the quantification of pERK1/2^T202/Y204^ and pp70S6K1^T389^ positive cells were counted due to an inhomogeneous staining pattern [50, 51]. An independent scoring system according to the H-Score was used to analyze pERK1/2^T202/Y204^, pAKT^S473^ and pp70S6K1^T389^ staining of the second patient cohort with newly diagnosed metastatic colorectal cancer as described previously [53]. In addition the immunhistochemical expression of PTEN was analyzed as described [52]. Baseline characteristics of the second cohort are summarized in Supplementary Table 4A.

For statistical analysis, chi-square test was used. Tumor DNA was isolated from FFPE tumor sections following microdissection. *KRAS* and *BRAF* mutation status was determined in the patient cohort treated with cetuximab and irinotecan by PCR amplification of the relevant exons followed by Sanger sequencing as described previously [52]. DNA from patients without *KRAS Exon 2* or *BRAF* mutations and DNA from the second patient cohort were analyzed by a 15-gene/62-exon panel read including exon 2-4 of *KRAS* and *NRAS*, Exon 15 of *BRAF* and exon 10 and 21 of *PIK3CA* on the MiSeq platform (Illumina, San Diego, CA) as described [52]. All studies of patient data or samples were approved by the Ethics Committee of the Medical Faculty of the University Duisburg-Essen (AZ.: 05-2882).

## SUPPLEMENTARY MATERIALS FIGURES AND TABLES


