# Supplementary material for: Molecular dissection of effector mechanisms of RAS-mediated resistance to anti-EGFR antibody therapy
Source: Oncotarget. 2017 Apr 26;8(28):45898–917. doi: 10.18632/oncotarget.17438 (PMC5542236; doi:10.18632/oncotarget.17438)
Supplement: Supplementary file 1 [file oncotarget-08-45898-s001.pdf]

Molecular dissection of effector mechanisms of *RAS*-mediated resistance to anti-EGFR antibody therapy

SUPPLEMENTARY MATERIALS

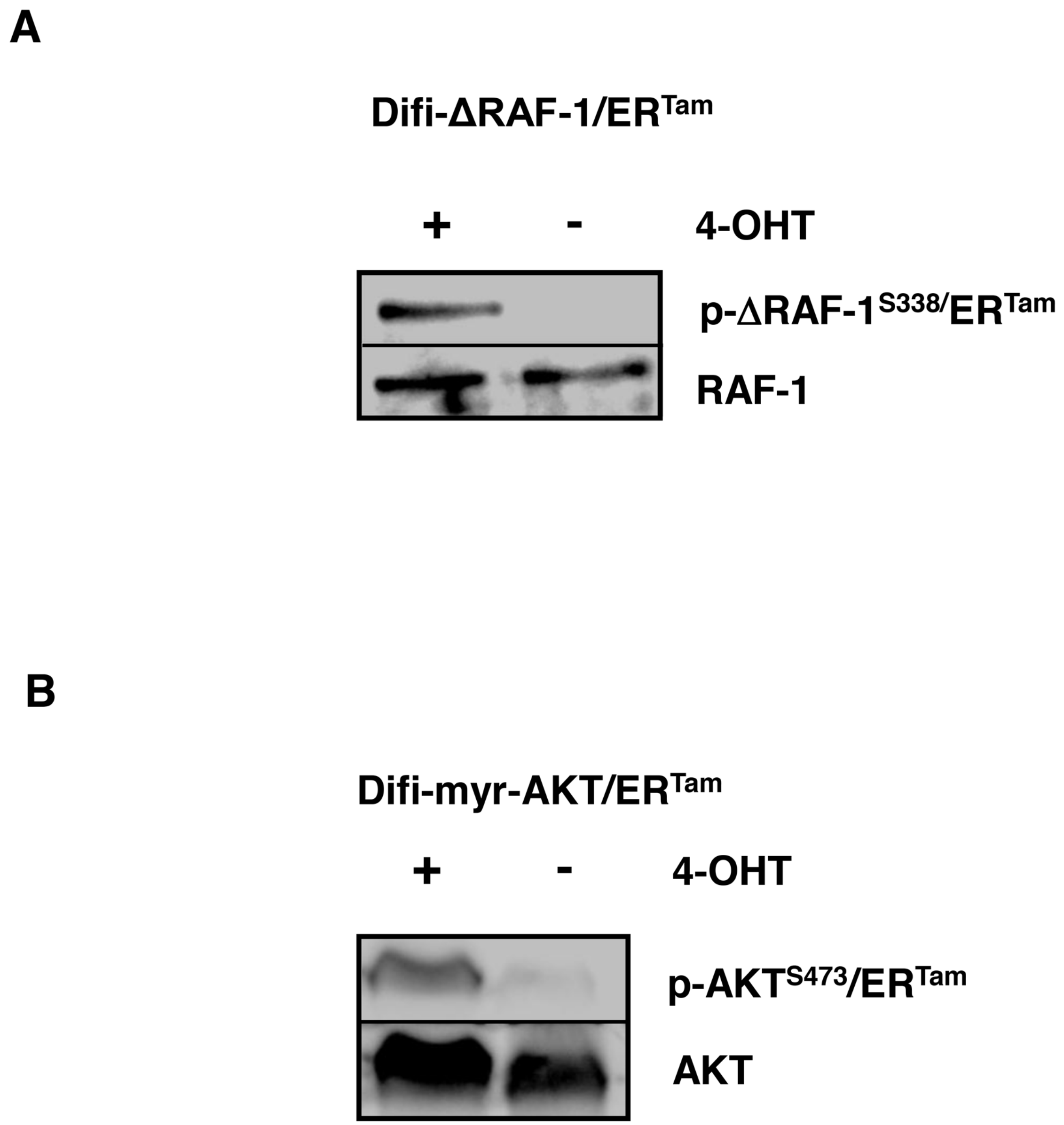

Supplementary Figure 1: (A) Difi *RAS* wild type cells were retrovirally transduced to stably express a ΔRAF-1/ER^{Tam} construct. Phosphorylation of ΔRAF-1/ER^{Tam} was strongly induced by the addition of 4-hydroxytamoxifen (4-OHT). **(B)** Difi *RAS* wild type cells were retrovirally transduced to stably express a myristoylated-AKT/ER^{Tam} (myr-AKT/ER^{Tam}) construct. Phosphorylation of myr-AKT/ER^{Tam} was strongly induced by the addition of 4-hydroxytamoxifen (4-OHT).

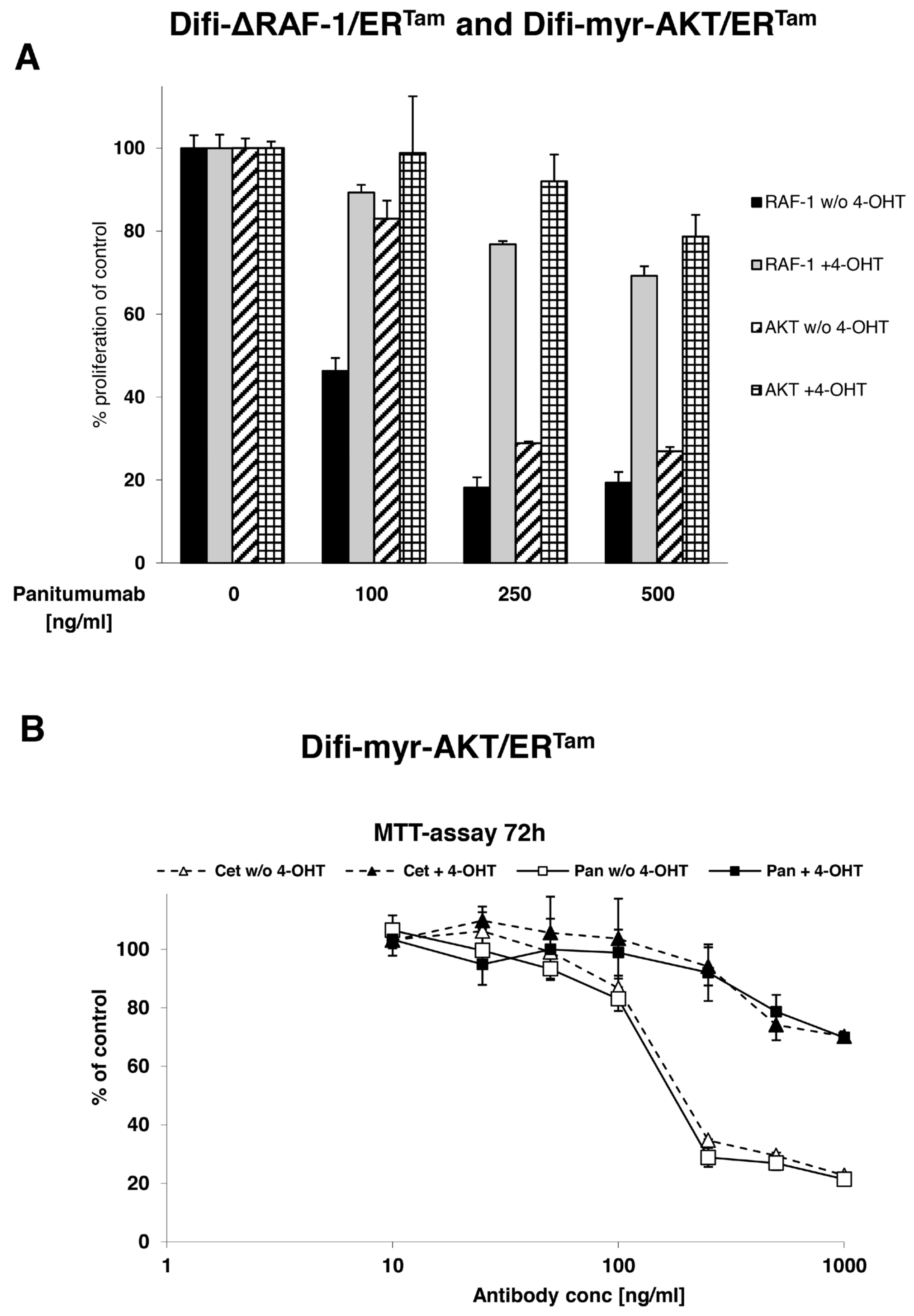

Supplementary Figure 2: (A) Clonogenic survival of Difi-ΔRAF-1/ER^{Tam}- and Difi-myr-AKT/ER^{Tam} cells cultured in the presence of panitumumab with or without 4-OHT as indicated. Mean colony numbers (+ SD) normalized to medium control from three independent experiments are given. **(B)** Proliferation of Difi-myr-AKT/ER^{Tam} cells grown in the presence of increasing concentrations of cetuximab or panitumumab with or without 4-OHT as indicated. Mean values (± SD) of three independent MTT assays.

A431-ΔRAF-1/ER^{Tam}

A p-ERK1/2$^{T202/Y204}$-staining

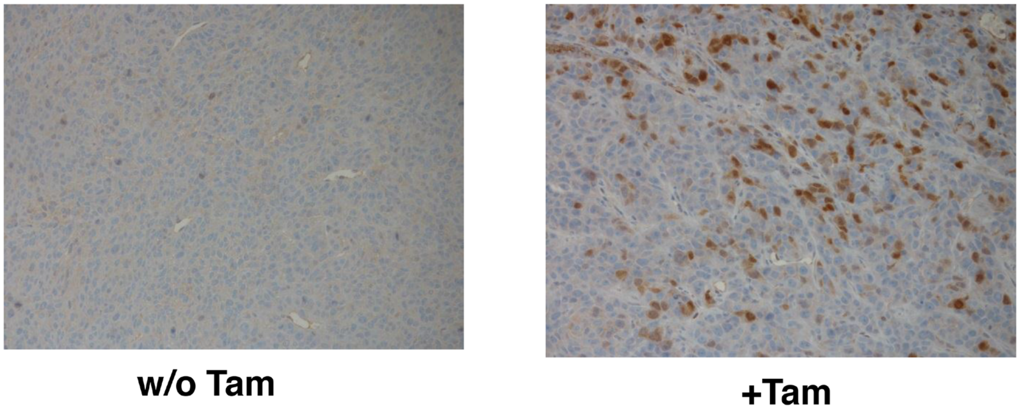

B

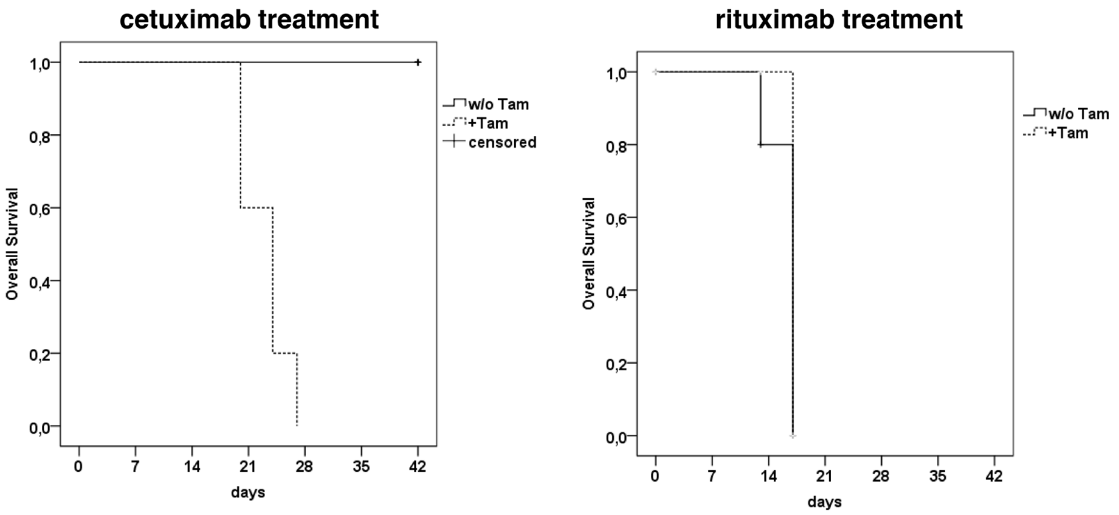

	median [days]	p-value (log rank)
w/o Tam	n.r	0.002
+ Tam	24	

	median [days]	p-value (log rank)
w/o Tam	17	0.317
+ Tam	17	

Supplementary Figure 3: (A) Representative immunhistochemical staining of p-ERK1/2$^{T202/Y204}$ in explanted A431-ΔRAF-1/ER^{Tam} tumors of NOD/SCID mice fed with or without tamoxifen diet. **(B)** Kaplan-Meier plots of overall survival of NOD/SCID mice following subcutaneous injection of 2x10^{6}A431-ΔRAF-1/ER^{Tam} cells. Mice were fed with (dashed line) or without (solid line) tamoxifen diet and were treated with 1 mg cetuximab (left) or 1 mg rituximab (right) twice weekly. Tamoxifen fed mice exhibited a significantly reduced overall survival upon cetuximab treatment as compared with mice fed without tamoxifen (p = 0.002, log rank test) (left). Upon rituximab treatment mice fed with or without tamoxifen diet died rapidly due to progressive tumor growth (right).

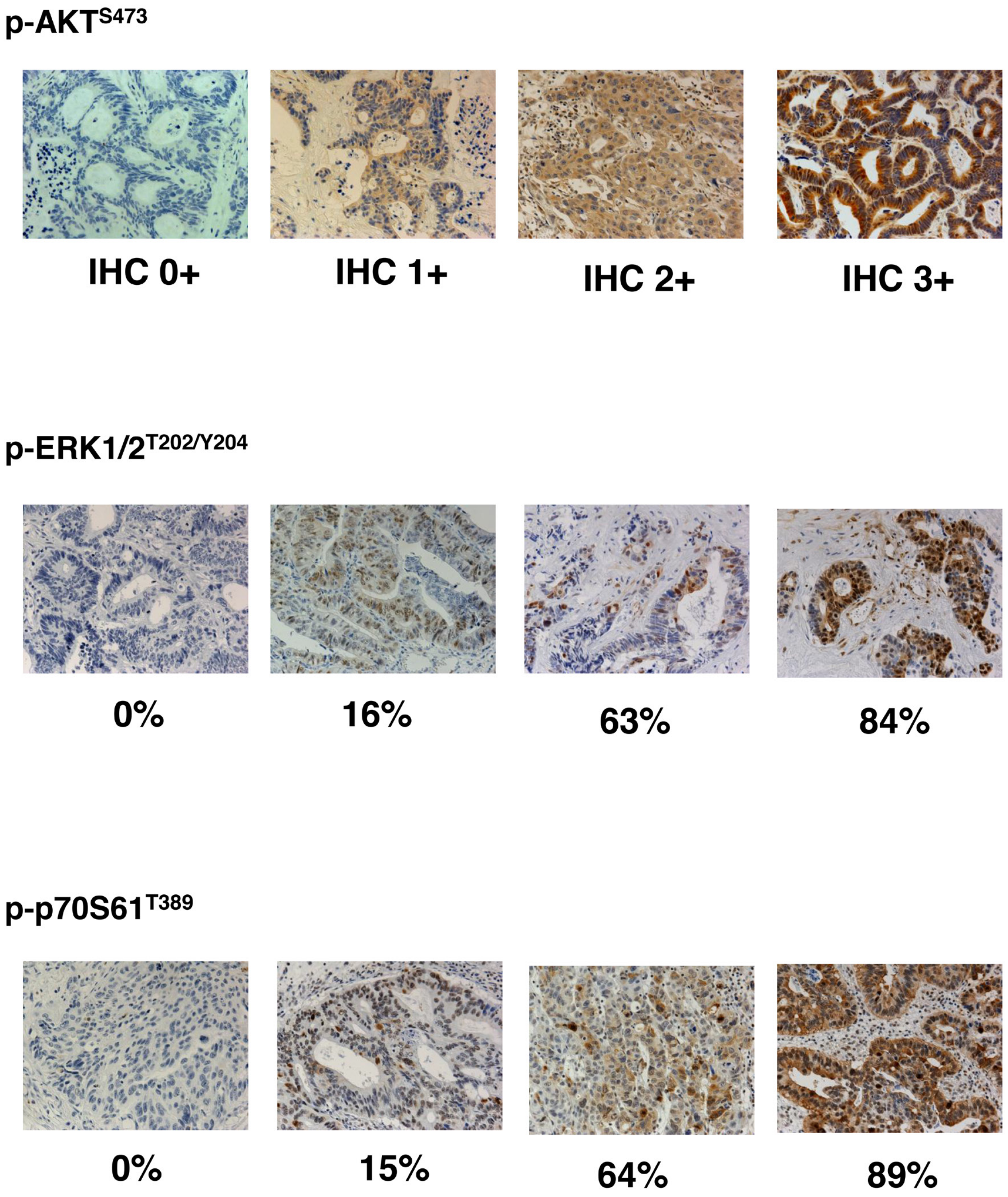

Supplementary Figure 4: Representative immunhistochemical stainings of p-AKT^{S473}. p-ERK1/2$^{T202/Y204}$ and p-p70S61^{T389} in FFPE tumor samples of patients with metastatic colorectal cancer treated with cetuximab and irinotecan.

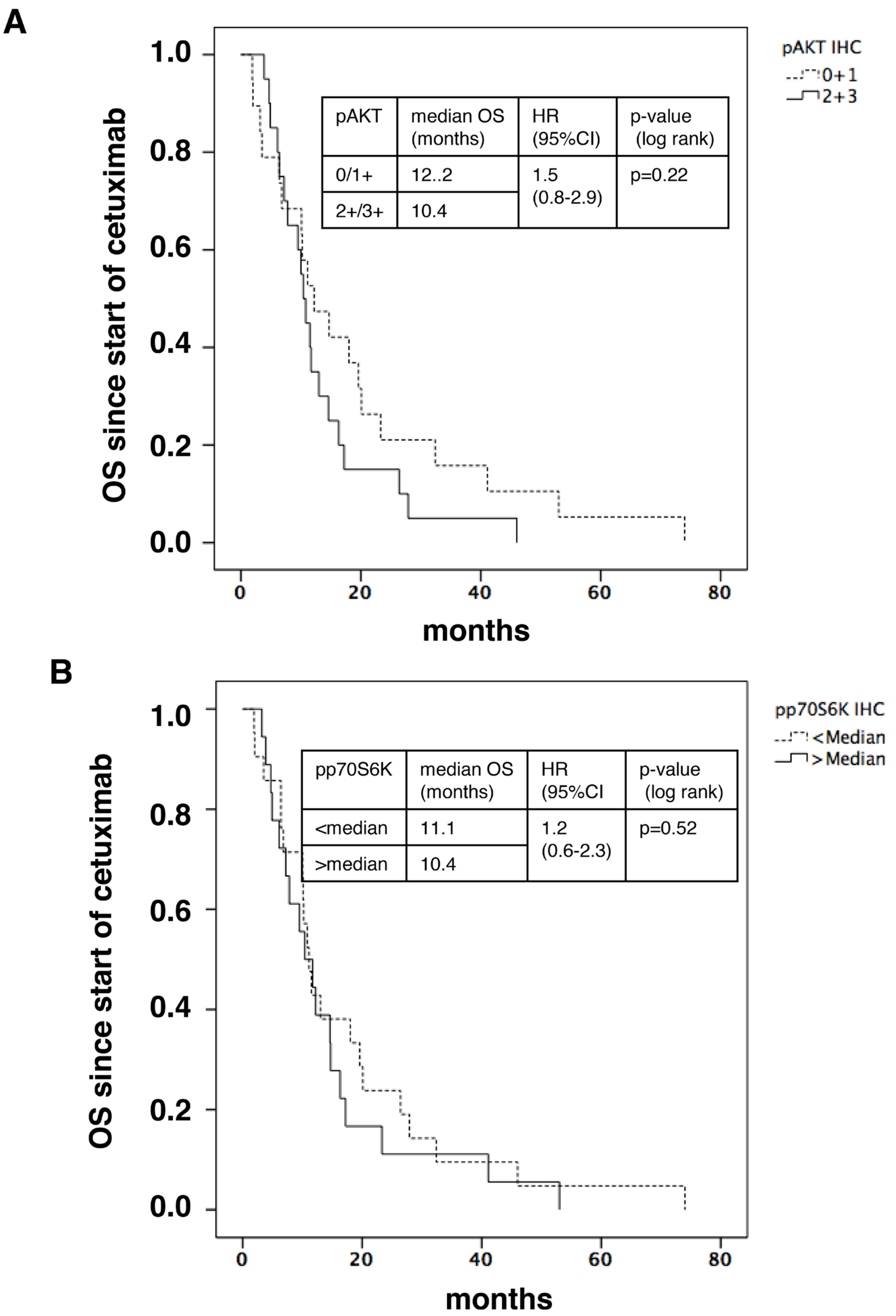

Supplementary Figure 5: (A) Kaplan-Meier plot of overall survival (OS) from start of treatment with cetuximab in combination with irinotecan of patients with mCRC with immunohistochemically low stained (IHC0+ and 1+) (dashed line) and strong stained (IHC2+ and3+) (solid line) pAKT^{S473}. Patients with tumors stained low for p-AKT^{S473} demonstrated a numerical prolonged OS (12.2 vs. 10.4 months; p = 0.22 log rank). **(B)** Kaplan-Meier plot of overall survival (OS) of patients with immunohistochemically stained p-p70S6K1^{T359} below the median (dashed line) or above the median (solid line). Patients with tumors stained for p-p70S6K1^{T359} below the median demonstrated a numerical prolonged OS (11.1 vs. 10.4 months; p = 0.52 log rank). The difference did not reach statistical significance.

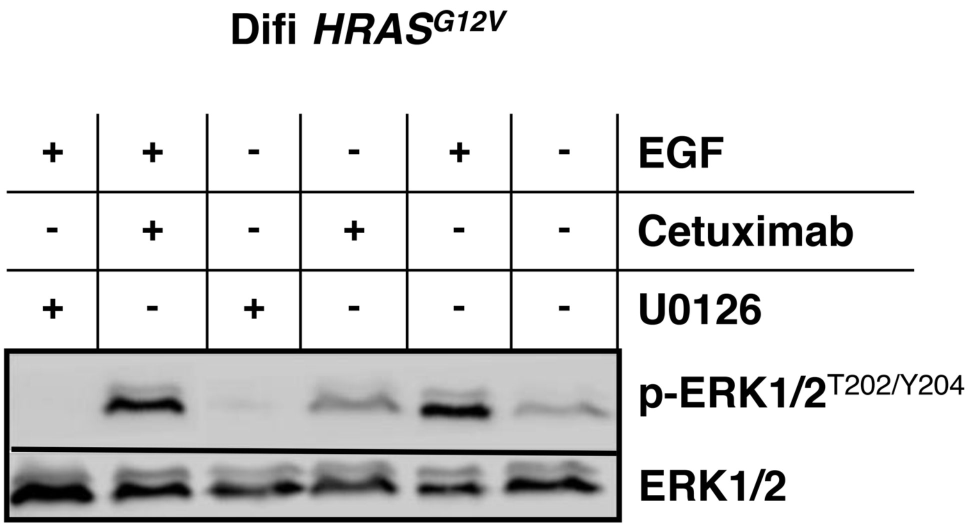

Supplementary Figure 6: Immunoblot analysis of Difi-HRAS^{G12V} cells treated with EGF (10 ng/ml), cetuximab (1 µg/ml), the MEK inhibitor U0126 (1 µM) and in combination. Inhibition of constitutive and ligand induced phosphorylation of MAPK-signal transducer ERK1/2 by the pharmacological inhibitor U0126. Note that cetuximab alone did not inhibit the phosphorylation of ERK1/2 in Difi-HRAS^{G12V} cells.

Supplementary Table 1: Patients characteristics (N = 39)

Characteristics	N (%)
Female	11 (28%)
Median age (range)	60 years (41-80)
Median time from diagnosis to start cetuximab/irinotecan (range)	20.5 months (6.5-77.4)
Tumor localization	
Rectum	10 (26%)
Colon	29 (74%)
Synchronous metastases	28 (72%)
Resection of primary tumor	35 (90%)
Adjuvant/neoadjuvant (radio-) chemotherapy	6 (15%)
Liver limited disease	20 (51%)
Median lines of chemotherapy	3 (1-5)
Irinotecan received	39 (100%)
Oxaliplatin received	37 (95%)
Fluoropyrimidin received	39 (100%)
Bevacizumab received	8 (20%)
VEGFR-TKI received (vatalanib, sorafenib)	13 (33%)
Mitomycin received	2 (5%)
KRAS exon 2 mutations (3 patients not tested)	14 (39%)
Codon 12	11 (31%)
Codon 13	3 (8%)
BRAF exon 15 mutations (5 patients not tested)	3 (9%)
Codon 600	3 (9%)
All RAS/BRAF tested population (N = 23)	
KRAS exon 2 mutations	15 (65%)
Codon 12	12 (52%)
Codon 13	3 (13%)
BRAF exon 15 mutations	3 (13%)
Codon 600	3 (13%)

Supplementary Table 2: Expression of pAKT^{S473}, pERK1/2$^{T202/Y204}$, and pp70S6K1^{T389} (N = 39)

Marker	N (%)
pAKT^{S473}	
0+	6 (15%)
1+	13 (33%)
2+	14 (36%)
3+	6 (15%)
pERK1/2$^{T202/Y204}$ median (range)	21% (0-84%)
pp70S6K1^{T389} median (range)	17.4% (9-90%)

Supplementary Table 3A: All *RAS*/*BRAF* population (N = 23)

All pts	pAKT^{S473} low	pAKT^{S473} high	pERK1/2$^{T202/Y204}$ ≤ median	pERK1/2$^{T202/Y204}$ > median
**pp70S6K1^{T389} < median**	9	6	12	3
**pp70S6K1^{T389} > median**	1	7	1	7
	p = 0.038, chi-square; odds ratio (95% CI): 10.500 (1.015-108.577)		p = 0.003, chi-square; odds ratio (95% CI): 28.000 (2.422-323.703)	
**pERK1/2$^{T202/Y204}$ ≤ median**	9	4		
**pERK1/2$^{T202/Y204}$ > median**	1	9		
	p = 0.006, chi-square; odds ratio (95% CI): 20.250 (1.878-218.390)			

Supplementary Table 3B: All *RAS*/BRAF population (N = 23)

	all RAS/BRAF wt		*RAS mut or BRAFmut*		*all RAS/BRAF wt*		*RAS mut or BRAFmut*	
	**pp70S6K1^{T389} ≤ median**	**pp70S6K1^{T389} > median**	**pp70S6K1^{T389} ≤ median**	**pp70S6K1^{T389} > median**	**pAKT^{S473} low**	**pAKT^{S473} high**	**pAKT^{S473} low**	**pAKT^{S473} high**
**pERK1/2$^{T202/Y204}$ ≤ median**	3	1	9	0	3	1	3	3
**pERK1/2$^{T202/Y204}$ > median**	0	2	3	5	0	2	1	7
	p = 0.2, chi-square; odds ratio (95% CI): 0.250 (0.046-1.365)[1]		p = 0.009, chi-square; odds ratio (95% CI): 2.667 (1.090-6,524)[2]		p = 0.200, chi-square; odds ratio (95% CI): 0.250 (0.046-1.365)[3]		p = 0.036, chi-square; odds ratio (95% CI): 14.000 (1.135-172.642)	

[1]For pp70S6K1^{T389} > median cohort; [2]for pp70S6K1^{T389} ≤ median cohort; [3]for pAKT^{S473} high cohort.

Supplementary Table 3C: Efficacy data (all *RAS*/*BRAF* population; N = 23)

	**N**	**%**
ORR	4	17
CR	0	0
PR	4	17
SD	13	57
PD	6	26
Median PFS	3.5 months (2.2-4.8)	
Median OS since start of cetuximab	10.4 months (9.3-11.5)	

(*Continued*)

Supplementary Table 3D: ORR (all *RAS*/BRAF population; N = 23)

Marker	ORR (%)	Odds ratio (95% CI)	p-value
All patients	17		
RAS wt vs *mut**	25 vs 13	2.2 (0.2-19.3)	0.48
BRAF wt vs *mut*#	21 vs 0	1.2 (1.0-1.5)	0.38
All RAS/BRAF wt vs *RAS or BRAF mut*	33 vs 12	3.8 (0.4-35.6)	0.23
pERK1/2$^{T202/Y204}$<median vs >median	23 vs 10	2.7 (0.2-30.8)	0.41
pAKT^{S473} 0+/1+ vs 2+/3+	30 vs 8	5.1 (0.4-59.5)	0.16
pp70S6K1^{T389}<median vs >median	20 vs 12	1.8 (0.2-20.2)	0.65
RAS/BRAF wt and pERK1/2$^{T202/Y204}$<median vs >median	25 vs 50	0.3 (0.0-11.9)	0.60
*RAS mut** or *BRAF mut*# and pERK1/2$^{T202/Y204}$<median vs >median	22 vs 0	2.1 (1.2-3.7)	0.16
RAS/BRAF wt and pAKT^{S473} 0+/1+ vs 2+/3+	33 vs 33	1.0 (0.0-29.8)	1.00
*RAS mut** or *BRAF mut*# and pAKT^{S473} 0+/1+ vs 2+/3+	39 vs 0	3.0 (1.5-6.1)	0.07
RAS/BRAF wt and pp70S6K1^{T389}<median vs >median	33 vs 33	1.0 (0.0-29.8)	1.00
*RAS mut** or *BRAF mut*# and pp70S6K1^{T389}<median vs >median	17 vs 0	1.5 (1.0-2.1)	0.33

*all *RAS*; #exon 15.

Supplementary Table 3E: PFS (all *RAS*/BRAF population; N = 23)

Marker	Median PFS (months)	HR (95% CI)	p-value (log rank)
All patients	3.5		
RAS wt vs *mut**	2.5 vs 3.5	0.8 (0.3-2.1)	0.68
BRAF wt vs *mut*#	3.5 vs 1.4	6.0 (1.1-25.8)	0.02
All RAS/BRAF wt vs *RAS or BRAF mut*	3.5 vs 3.3	1.3 (0.5-3.5)	0.65
pERK1/2$^{T202/Y204}$ < median vs > median	3.5 vs 2.8	0.7 (0.3-1.8)	0.44
pAKT^{S473} 0+/1+ vs 2+/3+	3.5 vs 3.5	0.6 (0.2-1.5)	0.27
pp70S6K1^{T389} < median vs > median	3.3 vs 4.1	0.4 (0.2-1.2)	0.09
RAS/BRAF wt and pERK1/2$^{T202/Y204}$ < median vs > median	3.5 vs 2.8	0.4 (0.0-3.9)	0.45
*RAS mut** or *BRAF mut*# and pERK1/2$^{T202/Y204}$ < median vs > median	3.5 vs 2.5	0.9 (0.3-2.6)	0.87
RAS/BRAF wt and pAKT^{S473} 0+/1+ vs 2+/3+	3.5 vs 4.2	0.2 (0.0-2.4)	0.23
*RAS mut** or *BRAF mut*# and pAKT^{S473} 0+/1+ vs 2+/3+	2.5 vs 3.3	0.8 (0.3-2.2)	0.61
RAS/BRAF wt and pp70S6K1^{T389} < median vs > median	3.5 vs 4.1	0.5 (0.1-3.2)	0.49
*RAS mut** or *BRAF mut*# and pp70S6K1^{T389} < median vs > median	3.3 vs 4.8	0.5 (0.1-1.5)	0.20

*all *RAS*; #exon 15.

(*Continued*)

Supplementary Table 3F: OS (all *RAS*/BRAF population; N = 23)

Marker	Median OS (months)	HR (95% CI)	p-value (log rank)
All patients	10.4		
RAS wt vs *mut**	10.8 vs 10.1	1.2 (0.5-2.9)	0.70
BRAF wt vs *mut*#	10.2 vs 10.8	1.5 (0.4-5.3)	0.53
All RAS/BRAF wt vs *RAS or BRAF mut*	10.4 vs 10.2	1.2 (0.5-3.1)	0.69
pERK1/2$^{T202/Y204}$ < median vs > median	11.5 vs 7.8	2.7 (1.1-7.0)	0.04
pAKT^{S473} 0+/1+ vs 2+/3+	10.2 vs 10.4	1.2 (0.5-3.0)	0.61
pp70S6K1^{T389} < median vs > median	10.8 vs 7.8	1.2 (0.5-2.9)	0.71
RAS/BRAF wt and pERK1/2$^{T202/Y204}$ < median vs > median	13.0 vs 4.9	5.8 (0.5-65.9)	0.15
*RAS mut** or *BRAF mut*# and pERK1/2$^{T202/Y204}$ < median vs > median	11.1 vs 7.8	2.3 (0.8-6.6)	0.13
RAS/BRAF wt and pAKT^{S473} 0+/1+ vs 2+/3+	20.1 vs 10.4	4.0 (0.4-39.5)	0.23
*RAS mut** or *BRAF mut*# and pAKT^{S473} 0+/1+ vs 2+/3+	10.2 vs 10.0	1.0 (0.4-2.7)	0.97
RAS/BRAF wt and pp70S6K1^{T389} < median vs > median	13.0 vs 10.4	0.8 (0.1-5.0)	0.83
*RAS mut** or *BRAF mut*# and pp70S6K1^{T389} < median vs > median	10.2 vs 7.8	1.5 (0.5-4.5)	0.48

*all *RAS*; #exon 15.

Supplementary Table 4A: Patients characteristics (N = 88)

Characteristics	N (%)
**Female**	39 (44%)
**Median age at profiling (range)**	58 years (23-83)
***KRAS* mutations**	22 (25%)
Exon 2	19 (22%)
Exon 3	2 (2%)
Exon 4	1 (1%)
***NRAS* mutations**	7 (8%)
Exon 2	2 (2%)
Exon 3	5 (6%)
Exon 4	0 (0%)
***All RAS* mutations**	29 (33%)
***BRAF* exon 15 mutation**	6 (7%)
***All RAF/BRAF* mutations**	35 (40%)
***PIK3CA* mutations**	7 (8%)
Exon 10	7 (8%)
Exon 21	0 (0%)

(*Continued*)

Supplementary Table 4B: Expression of pAKT^{S473}, pERK1/2$^{T202/Y204}$, pp70S6K1^{T389}, and PTEN

Marker	N (%)
**pAKT^{S473} H-Score (0-9)**	
0	28 (32%)
1	1 (1%)
2	8 (9%)
3	48 (55%)
4	1 (1%)
5	0 (0%)
6	2 (2%)
7	0 (0%)
8	0 (0%)
9	0 (0%)
**pERK1/2$^{T202/Y204}$ H-Score (0-9)**	
0	27 (31%)
1	22 (25%)
2	17 (19%)
3	7 (8%)
4	9 (10%)
5	0 (0%)
6	6 (7%)
7	0 (0%)
8	0 (0%)
9	0 (0%)
**pp70S6K1T389 H-Score (0-9)**	
0	2 (2%)
1	6 (7%)
2	6 (7%)
3	60 (68%)
4	5 (6%)
5	0 (0%)
6	8 (9%)
7	0 (0%)
8	0 (0%)
9	1 (1%)
**PTEN**	
Loss*	12 (14%)
Expressed	76 (86%)

*H-Score: 0 and 1.
